# Correlation analysis of AVPR1a and AVPR2 with abnormal water and sodium and potassium metabolism in rats

**DOI:** 10.1515/biol-2022-0949

**Published:** 2024-11-23

**Authors:** Kun Sun, Yingzhu Qiu, Yao Sun

**Affiliations:** Department of Nephropathy, Shandong Zibo Central Hospital, Zibo, 255036, Shandong, China; Department of Spine Surgery, Renji Hospital Affiliated to Shanghai Jiao Tong University, Shanghai, 200120, China; Department of General Practice, Shandong ZiBo Central Hospital, Zibo, 255036, Shandong, China

**Keywords:** arginine vasopressin receptor 1a, arginine vasopressin receptor 2, cardiorenal syndrome, abnormal metabolism of water and sodium

## Abstract

In clinical practice, an increasing number of patients exhibit concurrent cardiac and renal dysfunction, known as “cardiorenal syndrome,” where each condition exacerbates the other, resulting in poorer patient prognosis. Fluid and sodium retention can lead to excessive fluid overload in the body; therefore, correcting fluid and sodium metabolic disorders is crucial for alleviating patient symptoms. This study was to investigate the abnormalities in water and sodium metabolism, as well as the expression levels of arginine vasopressin receptor 1a (AVPR1a) and arginine vasopressin receptor 2 (AVPR2), in a rat model of chronic renal failure–chronic heart failure (CRF–CHF). One hundred male Sprague-Dawley rats were randomly assigned into four groups: the CG group (normal feeding), the CRF group (3/4 nephrectomy using a “two-step surgical method”), the CHF group (subcutaneous injection of isoproterenol at 100 mg/kg), and the CRF–CHF group (3/4 nephrectomy followed by a subcutaneous injection of isoproterenol at 100 mg/kg 1 week later). 4 weeks post-surgery, urine and blood samples were collected to measure 24 h urinary protein, sodium, and potassium levels. Serum creatinine (SCr) and blood urea nitrogen (BUN) levels were determined using assay kits. Left ventricular end diastolic pressure (LVEDP) and left ventricular systolic pressure (LVSP) were measured via left ventricular catheterization. The heart was weighed to calculate the left ventricular weight to body weight ratio (LVW/BW). The renal cortex and medulla were isolated to assess the relative mRNA and protein expression levels of AVPR1a and AVPR2. Compared to the CG group, the CRF and CRF–CHF groups exhibited significantly elevated levels of 24 h urinary protein, SCr, BUN, and relative expression levels of AVPR1a and AVPR2 in the renal cortex and medulla. The CHF and CRF–CHF groups showed significant increases in LVEDP and LVW/BW (*P* < 0.05). Additionally, compared to the CG group, the other three groups had significantly increased urinary sodium and blood potassium levels, and significantly decreased urinary potassium and blood sodium levels (*P* < 0.05). Compared to the CRF and CHF groups, the CRF–CHF group exhibited significantly higher levels of 24 h urinary protein, SCr, BUN, and relative expression levels of AVPR1a and AVPR2 in the renal cortex and medulla, along with significantly increased LVEDP and LVW/BW, significantly reduced LVSP, significantly increased urinary sodium and blood potassium levels, and significantly decreased urinary potassium and blood sodium levels (*P* < 0.05). Rats with CRF–CHF experienced exacerbated renal and cardiac failure, characterized by significant disturbances in water and sodium metabolism and abnormal expression of AVPR1a and AVPR2.

## 1 Introduction

Chronic renal failure–chronic heart failure (CRF–CHF) is a prevalent clinical condition, with its incidence increasing annually [1]. The kidneys serve as crucial excretory organs in the human body, primarily responsible for eliminating metabolic waste products and excess fluids to maintain water–electrolyte balance. Impaired renal function in CRF contributes to sodium and water retention to some extent [2], leading to fluid accumulation in the body, increased cardiac workload, and ultimately culminating in CHF [3]. Clinical manifestations of CRF–CHF include symptoms such as dyspnea, edema, fatigue, and palpitations, with severe cases possibly developing into complications like heart failure [4]. Current clinical management of CRF–CHF predominantly involves surgical interventions or pharmacotherapy. Key pharmacological treatments include diuretics, angiotensin-converting enzyme inhibitors, and angiotensin II receptor blockers, aimed at reducing sodium and water retention to lower blood pressure and improve cardiac function [5]. Surgical treatments such as coronary artery bypass grafting or implantation of cardiac pacemakers are utilized to enhance cardiac function and alleviate cardiac burden in patients.

The renin–angiotensin–aldosterone system (RAAS) is a critical mechanism regulating blood pressure, water, and electrolyte balance, and it also plays a role in the regulation of blood pressure and sodium–water retention [6]. Arginine vasopressin receptor 1a (AVPR1a) and arginine vasopressin receptor 2 (AVPR2) are two receptors associated with RAAS [7,8]. AVPR1a is predominantly expressed in locations such as the adrenal cortex, where it functions to constrict blood vessels, raise blood pressure, and promote aldosterone secretion from the adrenal cortex [9]. AVPR2, on the other hand, is primarily located in the epithelial cells of renal tubules and facilitates water reabsorption in the kidney tubules [10]. Activation of AVPR2 also promotes sodium reabsorption in the renal tubules, thereby increasing blood volume and blood pressure. Recent studies indicated that dysregulation of AVPR1a and AVPR2 expression can lead to various diseases. Overactivation of AVPR1a may contribute to conditions such as hypertension, heart failure, and hyperfunction of the adrenal cortex [9]. Defects in AVPR2 can lead to nephrogenic diabetes insipidus, characterized by increased urine volume, diluted urine, and reduced blood volume [11].

Patients with CRF experience impaired renal tubular and collecting duct reabsorption functions, resulting in varying degrees of electrolyte and water imbalance [12]. This disturbance further affects the roles of AVPR1a and AVPR2. However, there is currently a lack of research investigating the correlation between AVPR1a, AVPR2, and the abnormalities in water and sodium metabolism in CRF–CHF patients. Therefore, to address this gap, this study aimed to establish CRF, CHF, and CRF–CHF rat models to examine renal function, cardiac function, water–sodium metabolism, and changes in AVPR1a and AVPR2 expression. The goal was to elucidate the relationship between AVPR1a, AVPR2, and water–sodium metabolism abnormalities in CRF–CHF, providing insights for future related research endeavors.

## 2 Methods and materials

### 2.1 Experimental animals

A total of 100 male cleaning grade Sprague-Dawley rats (6–8 weeks old) weighing 200–250 g were randomly divided into four groups, namely normal CG (*n* = 25), CRF (*n* = 25), CHF (*n* = 25), and CRF–CHF (*n* = 25) groups. Before the commencement of the experiment, all animals were housed in an animal laboratory maintained at a temperature of 22–25°C and a relative humidity of 45–55%, with a 12 h light/dark cycle. The animals had free access to food and water and were acclimatized to the laboratory environment for 1 week prior to the start of the study.


**Ethical approval:** The research related to animal use has been complied with all the relevant national regulations and institutional policies for the care and use of animals, and has been approved by the Medical Committee of Zibo Central Hospital, Shandong Province.

### 2.2 Experimental design

The CG rats received no treatment, the CRF rats were used to establish the CRF model, the CHF rats were used to establish the CHF model, and the CRF–CHF rats were used to establish the CHF model on the basis of the CRF model. All rats, including the CG group, were fasted for 12 h prior to surgery. Four weeks post-surgery, cardiac and renal function changes were analyzed. Urine and blood samples were collected to measure urinary sodium, urinary potassium, serum sodium, and serum potassium levels. At the end of the experiment, the rats were euthanized, and their kidneys were harvested. Real-time quantitative polymerase chain reaction and western blotting were performed to detect the mRNA and protein levels of AVPR1a and AVPR2 in the renal cortex and medulla. This experiment was approved by the Medical Committee of Zibo Central Hospital, Shandong Province, and the study protocol adhered to ethical standards.

### 2.3 Preparation of animal models

The CRF group rats underwent a “two-step surgery” to establish the CRF model [13]. Rats were anesthetized with an intraperitoneal injection of 30 mg/kg of 0.3% pentobarbital sodium, and then fixed in a supine position on the surgical table. After routine disinfection, a midline abdominal incision was made, and the kidneys were exposed by layer-by-layer dissection. In the first week, the lower pole (1/2) of the left kidney was removed, and 1 week later, a total nephrectomy of the right kidney was performed.

The CHF group rats were induced with isoproterenol to establish the CHF model. They received subcutaneous injections of isoproterenol at 100 mg/kg (administered in two doses, with a 24 h interval between injections).

For the CRF–CHF group, 1 week after establishing the CRF model using the “two-step surgery,” the rats received subcutaneous injections of isoproterenol at 100 mg/kg (administered in two doses, with a 24 h interval between injections) to induce the CRF–CHF model.

The CG group rats underwent two laparotomies without kidney removal.

All rats that underwent surgery were routinely sutured and disinfected postoperatively, housed separately, and monitored for changes in vital signs. No mortality was observed in any group.

### 2.4 Sample collection

Four weeks post-surgery, samples were collected. The rats were placed in metabolic cages to collect 24 h urine samples. Blood samples were obtained via the tail vein. Twenty-four hours after blood collection, left ventricular function and hemodynamic parameters were assessed. Subsequently, the rats were anesthetized with an intraperitoneal injection of 30 mg/kg of 0.3% pentobarbital sodium. After body weight was measured, the rats were euthanized, and their hearts and kidneys were harvested. The organs were rinsed to remove excess blood, blotted dry, and weighed. The kidneys were sectioned along the coronal plane, and the renal cortex and medulla were separated, cut into small pieces, and stored at −80°C for further analysis.

### 2.5 Detection methods

#### 2.5.1 Renal function testing

The 24 h urinary protein content was measured using the Bradford method. Serum creatinine (SCr) and blood urea nitrogen (BUN) levels were determined according to the instructions provided in the enzyme-linked immunosorbent assay kit.

#### 2.5.2 Cardiac function testing

Hemodynamic parameters, including left ventricular end diastolic pressure (LVEDP) and left ventricular systolic pressure (LVSP), were measured using left ventricular catheterization via the left common carotid artery. The catheter was connected to a BL-420F biological function experiment system (Chengdu Taimeng Technology Co., Ltd, China). After the measurements were completed, heart tissue was isolated to determine the left ventricular weight (LVW). The extent of left ventricular remodeling was assessed by calculating the LVW to body weight (BW) ratio.

#### 2.5.3 Detection of water and sodium metabolism

The sodium and potassium levels in rat blood were measured using the Chiron 348 blood chemistry analyzer (Chiron Diagnostics Corp., USA). The sodium and potassium levels in 24 h urine samples were measured using the electrode of the Animal-550 fully automatic electrolyte analyzer (Shanghai Huanxi Medical Equipment Co., Ltd, China).

#### 2.5.4 Real-time fluorescence quantitative PCR (qPCR) detection

Approximately 200 mg of renal cortex and medulla tissue were homogenized in Trizol reagent for total RNA extraction. The PrimeScript™ RT reagent kit (Perfect Real Time) was used for reverse transcription to synthesize cDNA according to the manufacturer’s instructions. The synthesized cDNA was used as a template for fluorescent qPCR using the TB Green^®^ Premix Ex Taq™ II (Tli RNaseH Plus) Bulk kit. The PCR amplification program consisted of 95°C for 30 s, followed by 40 cycles of 95°C for 5 s and 60°C for 30 s. The primers used for amplification were as follows: for AVPR1a, forward 5′-GCTGGCGGTGATTTTCGTG-3′ and reverse 5′-GCAAACACCTGCAAGTGCT-3′; for AVPR2, forward 5′-GCTGTGGCTCTGTTTCAAGTG-3′ and reverse 5′-CCAGGATCATGTAGGAAGAGGC-3′; for GAPDH, forward 5′-AATGGATTTGGACGCATTGGT-3′ and reverse 5′-TTTGCACTGGTACGTGTTGAT-3′. GAPDH was used as an internal reference gene, and the relative expression levels of the target genes AVPR1a and AVPR2 were calculated using the 2^−ΔΔCt^ method.

#### 2.5.5 Western blotting

Fifty milligrams of renal cortex and medulla tissue were homogenized in RIPA lysis buffer to extract total protein. The protein concentration was determined using a BCA Protein Assay Kit (Shanghai Beyotime Biotechnology Co., Ltd, China) according to the manufacturer’s instructions. Thirty micrograms of protein were loaded onto a sodium dodecyl sulfate-polyacrylamide gel electrophoresis gel, followed by transfer onto a polyvinylidene difluoride membrane. The membrane was blocked with 5% skim milk blocking solution at room temperature for 1 h. Rabbit polyclonal antibodies against AVPR1a (1:1,000; Abcam, UK), rabbit polyclonal antibodies against AVPR2 (1:1,000; Abcam, UK), and mouse monoclonal antibodies against β-actin (1:2,000; Abcam, UK) were added and incubated overnight at 4°C. After membrane washing, the secondary antibody, horseradish peroxidase-conjugated rabbit anti-mouse IgG antibody (1:5,000), was added and incubated at room temperature for 1 h. The membrane was washed again and subjected to enhanced chemiluminescence (Shanghai Beyotime Biotechnology Co., Ltd, China) for visualization, and the images were captured using a WD-9413D Gel Imaging System (Beijing Liuyi, China). β-actin was used as an internal control, and the relative expression levels of the target proteins AVPR1a and AVPR2 were calculated using *ImageJ*.

#### 2.5.6 Statistical methods

The measurement data were statistically analyzed using *SPSS 22.0*. All data were presented as mean ± standard deviation (*x̅* ± *s*). One-way analysis of variance was performed, and pairwise comparisons between groups were conducted using the LSD-*t* test. A *P*-value <0.05 was considered statistically significant.

## 3 Results

### 3.1 Comparison of renal function among different groups of rats

**Figure 1:**
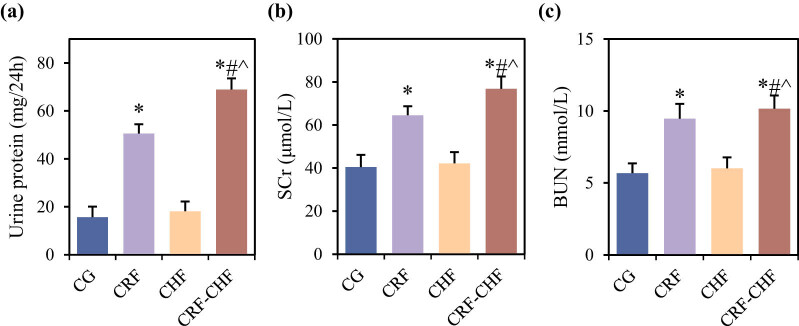
Comparison of renal function indicators among four groups of rats: (a) 24 h urine protein, (b) SCr, and (c) BUN. Compared with the CG group, *P < 0.05; compared with the CRF group, #*P* < 0.05; compared with the CHF group, ^*P* < 0.05.

Differences in renal function-related indicators including 24 h urinary protein, SCr, and BUN levels among the four groups of rats are compared in Figure 1. Compared to the CG group, rats in the CRF and CRF–CHF groups exhibited significantly elevated levels of 24 h urinary protein, SCr, and BUN (*P* < 0.05). Moreover, compared to the CRF and CHF groups, rats in the CRF–CHF group showed significantly higher levels of 24 h urinary protein, SCr, and BUN (*P* < 0.05).

### 3.2 Comparison of cardiac function among different groups of rats

**Figure 2:**
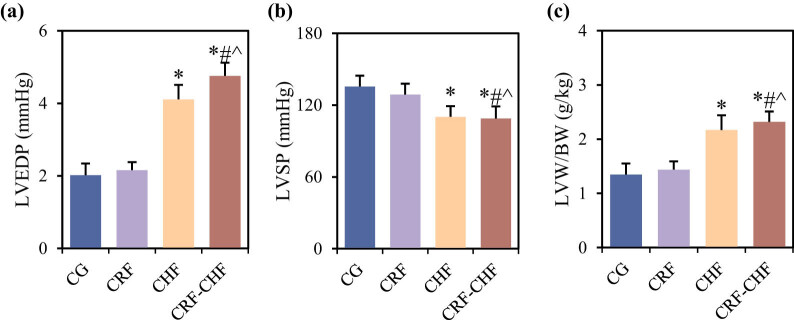
Comparison of cardiac function indicators among four groups of rats: (a) LVDEP, (b) LVSP, and (c) LVW/BW. Compared with the CG group, *P < 0.05; compared with the CRF group, #*P* < 0.05; compared with the CHF group, ^*P* < 0.05.

Differences in cardiac function-related indicators, including LVEDP, LVSP, and LVW/BW levels among the four groups of rats, are compared in Figure 2. Compared to the CG group, rats in the CHF and CRF–CHF groups exhibited significantly elevated levels of LVEDP and LVW/BW, and significantly decreased levels of LVSP (*P* < 0.05). Furthermore, compared to the CRF and CHF groups, rats in the CRF–CHF group showed significantly higher levels of LVEDP and LVW/BW, and significantly lower levels of LVSP (*P* < 0.05).

### 3.3 Comparison of water and sodium metabolism status among different groups of rats

**Figure 3:**
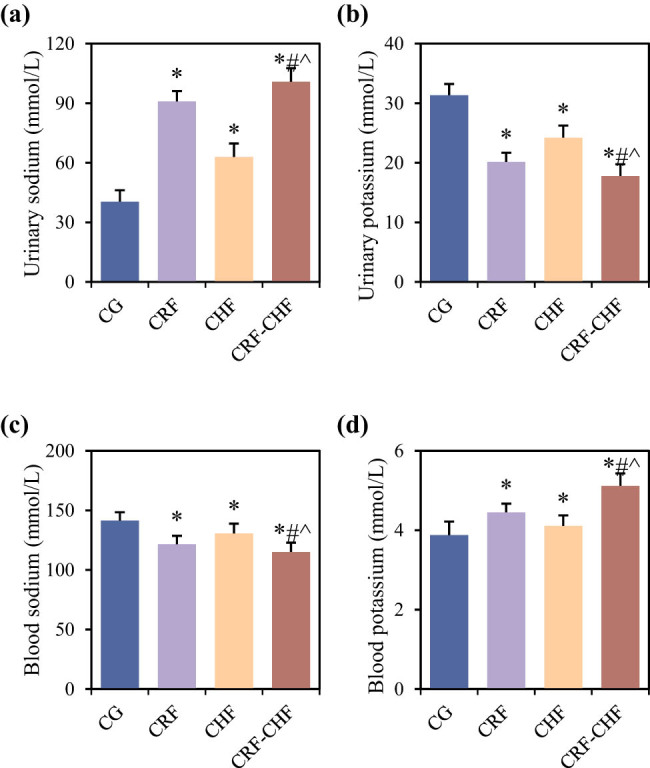
Comparison of water and sodium metabolism indicators among four groups of rats: (a) urine sodium content, (b) potassium content in urine, (c) blood sodium content, and (d) blood potassium content. Compared with the CG group, *P < 0.05; compared with the CRF group, #*P* < 0.05; compared with the CHF group, ^*P* < 0.05.

Differences in water and sodium metabolism-related indicators, including sodium and potassium levels among the four groups of rats, are compared in Figure 3. Compared to the CG group, rats in the CRF, CHF, and CRF–CHF groups exhibited significantly elevated levels of urinary sodium and blood potassium, while urinary potassium and blood sodium levels were significantly decreased (*P* < 0.05). Furthermore, compared to the CRF and CHF groups, rats in the CRF–CHF group showed significantly higher levels of urinary sodium and blood potassium, while urinary potassium and blood sodium levels were significantly decreased (*P* < 0.05).

### 3.4 Comparison of AVPR1a and AVPR2 expression levels in renal cortex and medulla of rats in each group

**Figure 4:**
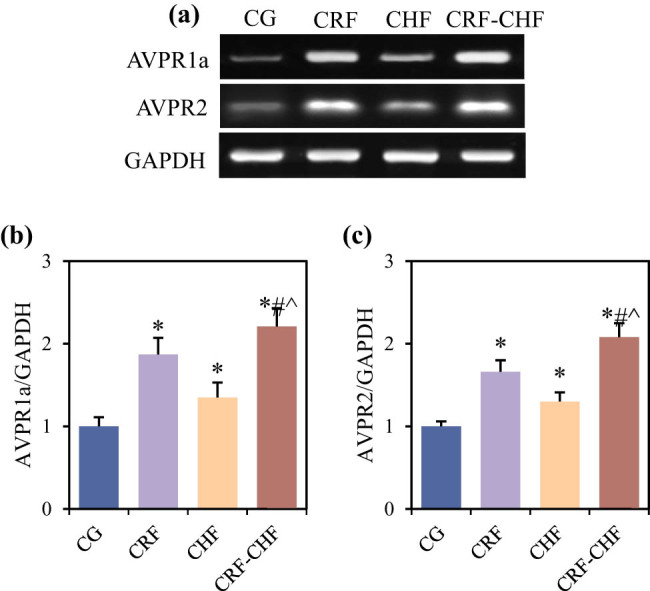
Comparison of relative expression levels of AVPR1a and AVPR2 mRNA in the renal cortex and medulla of four groups of rats: (a) PCR detection, (b) relative expression level of AVPR1a mRNA, and (c) relative expression level of AVPR2 mRNA. Compared with the CG group, *P < 0.05; compared with the CRF group, #*P* < 0.05; compared with the CHF group, ^*P* < 0.05.

Differences in the relative expression levels of AVPR1a and AVPR2 mRNA in the renal cortex and medulla among the four groups of rats are compared in Figure 4. Compared to the CG group, rats in the CRF, CHF, and CRF–CHF groups exhibited significantly elevated levels of AVPR1a and AVPR2 mRNA in the renal cortex and medulla (*P* < 0.05). Furthermore, compared to the CRF and CHF groups, rats in the CRF–CHF group showed significantly higher levels of AVPR1a and AVPR2 mRNA in the renal cortex and medulla (*P* < 0.05).

**Figure 5:**
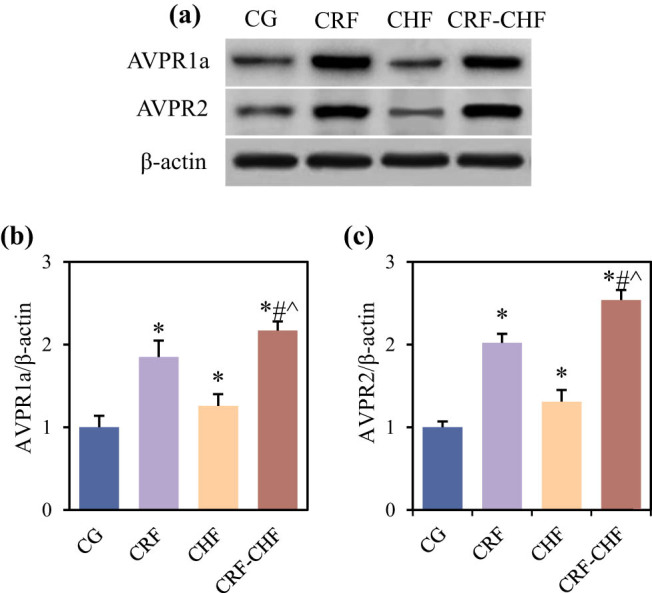
Comparison of relative expression levels of AVPR1a and AVPR2 proteins in the renal cortex and medulla of four groups of rats: (a) western blotting detection, (b) relative expression level of AVPR1a protein, and (c) relative expression level of AVPR2 protein. Compared with the CG group, *P < 0.05; compared with the CRF group, #*P* < 0.05; compared with the CHF group, ^*P* < 0.05.

Differences in the relative expression levels of AVPR1a and AVPR2 proteins in the renal cortex and medulla among the four groups of rats are compared in Figure 5. Compared to the CG group, rats in the CRF, CHF, and CRF–CHF groups exhibited significantly elevated levels of AVPR1a and AVPR2 proteins in the renal cortex and medulla (*P* < 0.05). Furthermore, compared to the CRF and CHF groups, rats in the CRF–CHF group showed significantly higher levels of AVPR1a and AVPR2 proteins in the renal cortex and medulla (*P* < 0.05).

## 4 Discussion

Hypertension, diabetes mellitus, and atherosclerosis are common underlying diseases in patients with heart failure and renal dysfunction [14,15]. Renal dysfunction is an independent predictor of heart failure [16]. Cardiac dysfunction is a severe complication of CRF and is closely associated with patient mortality [17]. In this study, a CRF rat model was prepared using a 3/4 nephrectomy procedure, and a CHF rat model was induced by subcutaneous injection of isoproterenol. The CRF–CHF rat model was established by combining the 3/4 nephrectomy procedure and subcutaneous injection of isoproterenol. The results showed that the levels of 24 h urinary protein, SCr, and BUN significantly increased in both CRF and CRF–CHF rats, while LVEDP and LVW/BW significantly increased, and LVSP significantly decreased in CHF and CRF–CHF rats. These findings indicated successful establishment of CRF, CHF, and CRF–CHF rat models. In addition, this study found that compared to CRF rats, CRF–CHF rats exhibited significantly increased levels of 24 h urinary protein, SCr, and BUN. Furthermore, compared to CHF rats, CRF–CHF rats showed significantly increased levels of LVEDP and LVW/BW, and significantly decreased levels of LVSP. Numerous studies confirmed that heart failure and renal failure often coexist, and patients with CRF commonly exhibit left ventricular hypertrophy, leading to the progression of “cardiorenal syndrome” [18,19]. When heart failure occurs, the cardiac output decreases, resulting in reduced renal perfusion and consequent pre-renal dysfunction. Reduced renal perfusion leads to renal ischemia, which in turn reduces erythropoietin levels, causing anemia. The reduced oxygen-carrying capacity of the blood exacerbates cardiac workload, induces cardiomyocyte apoptosis, and ultimately leads to cardiac functional impairment [20].

Under normal conditions, excessive intake of fluids stimulates the secretion of diuretics and inhibits the secretion of hormones involved in water and sodium reabsorption, thereby maintaining normal fluid balance and preventing the occurrence of edema. Sodium and water retention can lead to edema, hypertension, and heart failure [21,22]. Prolonged and severe sodium and water retention can exacerbate cardiac workload, disrupt normal blood circulation, and may even lead to the development of chronic congestive heart failure [23]. Currently, the activation of the RAAS, abnormal sympathetic nervous system activity, and renal dysfunction are considered important factors contributing to sodium and water retention [15,24,25]. In this study, compared to normal rats, rats with CRF, CHF, and CRF–CHF exhibited significantly increased levels of urinary sodium and blood potassium, while urinary potassium and blood sodium levels were significantly decreased. Additionally, it was observed that CRF–CHF rats had the highest levels of urinary sodium and blood potassium, and the lowest levels of urinary potassium and blood sodium. Hyponatremia is closely associated with water and sodium metabolism. Patients with heart failure experience impaired cardiac contraction and relaxation due to reduced cardiac function, leading to decreased cardiac output, venous return obstruction, and subsequent water and sodium retention [26]. Patients with renal failure experience reduced renal excretory function due to renal insufficiency, leading to an inability to properly excrete water and sodium from the body, resulting in water and sodium retention [27]. The phenomenon of water and sodium retention is exacerbated in patients with combined heart and kidney failure. AVPR1a and AVPR2 are two critical vasopressin receptors that play crucial roles in the human body, mainly regulating water and sodium metabolism [28]. AVPR1a is primarily distributed in the epithelial cells of renal tubules and collecting ducts, promoting water reabsorption in renal tubules, increasing urinary sodium excretion, and thereby maintaining body water balance [29]. AVPR2 is mainly distributed in the epithelial cells of renal glomeruli and distal convoluted tubules, promoting an increase in glomerular filtration rate and urine volume, thereby assisting the body in excreting excess water [30]. If the expression of AVPR1a and AVPR2 is abnormal, it will lead to disturbances in water and sodium metabolism, thus triggering a series of health problems. This study found that compared to normal rats, rats with CRF, CHF, and CRF–CHF exhibited significantly increased relative expression levels of AVPR1a and AVPR2 mRNA and proteins in the renal cortex and medulla. The CRF–CHF rats showed the highest relative expression levels of AVPR1a, AVPR2 mRNA, and proteins in the renal cortex and medulla. Due to the dual damage of heart failure and renal failure, CRF–CHF rats showed significant disturbances in sodium and water metabolism, and additionally, abnormal upregulation of AVPR1a and AVPR2 expression levels. Therefore, it can be inferred that AVPR1a and AVPR2 may be important regulatory factors in the sodium and water metabolism abnormalities observed in CRF–CHF rats.

## 5 Conclusion

The exacerbation of cardiac and renal dysfunction in CRF–CHF, along with significant disturbances in sodium and water metabolism, as well as the abnormal upregulation of AVPR1a and AVPR2 expression levels in the renal medulla, suggests that AVPR1a and AVPR2 may be involved in regulating the abnormal sodium and water metabolism in CRF–CHF. However, it is important to note that this article only provides a preliminary exploration of the correlation between AVPR1a, AVPR2, and the abnormal sodium and water metabolism in CRF–CHF rats. Further validation of these results is required in future studies. Additionally, clinical research is needed to investigate the roles of AVPR1a and AVPR2 in the pathogenesis of CRF–CHF. This finding provides new insights and directions for in-depth research into the pathogenesis of CRF–CHF, the identification of potential therapeutic targets, and the development of novel therapeutic drugs.
